# Controversial Issues in Kyphoplasty and Vertebroplasty in Osteoporotic Vertebral Fractures

**DOI:** 10.1155/2014/934206

**Published:** 2014-03-04

**Authors:** Ioannis D. Papanastassiou, Andreas Filis, Maria A. Gerochristou, Frank D. Vrionis

**Affiliations:** ^1^H. Lee Moffitt Cancer Center & Research Institute, Neuro-Oncology Program and Department of NeuroOncology and Orthopaedics, University of South Florida College of Medicine, 12902 Magnolia Drive, Tampa, FL 33647, USA; ^2^General Oncological Hospital Kifisias “Agioi Anargyroi”, Athens, Greece; ^3^“Andreas Syngros” Hospital, 16121 Athens, Greece

## Abstract

Kyphoplasty (KP) and vertebroplasty (VP) have been successfully employed for many years for the treatment of osteoporotic vertebral fractures. The purpose of this review is to resolve the controversial issues raised by the two randomized trials that claimed no difference between VP and SHAM procedure. In particular we compare nonsurgical management (NSM) and KP and VP, in terms of clinical parameters (pain, disability, quality of life, and new fractures), cost-effectiveness, radiological variables (kyphosis correction and vertebral height restoration), and VP versus KP for cement extravasation and complications profile. Cement types and optimal filling are analyzed and technological innovations are presented. Finally unipedicular/bipedicular techniques are compared. *Conclusion*. VP and KP are superior to NSM in clinical and radiological parameters and probably more cost-effective. KP is superior to VP in sagittal balance improvement and cement leaking. Complications are rare but serious adverse events have been described, so caution should be exerted. Unilateral procedures should be pursued whenever feasible. Upcoming randomized trials (CEEP, OSTEO-6, STIC-2, and VERTOS IV) will provide the missing link.

## 1. Introduction

Osteoporosis is considered to be amongst the 10 most important world diseases according to World Health Organization, leading to Vertebral Compression Fractures (VCFs) which dramatically increase morbidity and mortality [1–3]. Two randomized trials in the New England Journal of Medicine (NEJM) that showed no benefit from vertebroplasty (VP) over a simulated procedure [4, 5] may have contributed to the decline in utilization of Vertebral Augmentation Procedures (VAPs) and especially VP for treating VCFs in recent years [6, 7]. However, the field is expanding and there seems to be a tremendous scientific interest in VAPs, resulting in more than 250 articles published annually on VP and kyphoplasty (KP). Many issues remain controversial and even scientific societies give contradicted recommendations with interventional radiologists praising and orthopedic surgeons condemning VP [8, 9].

In view of this controversy we published a meta-analysis on comparative prospective trials in 2012 [10]. In this updated review we try to give insight into many of the questions in this analysis: (1) what is the evidence on pain relief/Quality of Life (QoL) improvement for VAPs versus NSM, (2) are VAPs cost-effective, (3) does KP provide more kyphotic reduction than VP and does this have a clinical implication, (4) is cement leaking more with VP, (5) what is the risk for adjacent fractures, (6) complication profile of VAPs, (7) cement types, characteristics, and optimal filling, (8) unipedicular versus bipedicular approach, and (9) newer designs.

## 2. Pain Relief/Disability/QoL Improvement

With the exception of the 2 NEJM RCTs and a smaller RCT that reported no benefit of VP versus SHAM [4, 5] or NSM [11, 12] the vast majority of prospective comparative studies (Tables 1 and 2) support the superiority of VAPs versus NSM [13–27], with only 1 newer study reporting better results from KP until the 1st postoperative month and then equivalence [28]. When comparing VP with KP, it seems that both procedures offer a 4-5 point average reduction from baseline (in a 10-point scale), so most authors believe that the threshold for performing the procedure is a preoperative Visual Analogue Scale (VAS) pain score of at least 4 or 5 [29–40]. Few studies report better pain relief from KP up to 1-2 years postoperatively [41, 42]. Patients in more severe pain seem to benefit most from the procedure [43].

In terms of disability/QoL improvement, the literature suggests that there may be an advantage of KP over VP and NSM [10]. Published studies either report equivalence of procedures [29, 30, 32, 34–40, 46] or superiority of KP [31, 41, 42]. Since those parameters may better reflect the efficacy of the procedures compared to pain scales (i.e., VAS), they should be strictly scrutinized in future trials; indeed pain measurements frequently represent variations (pain with or without medications, positional/maximal/nocturnal/average pain, etc.) and not surprisingly even between RCTs there are striking differences (i.e., double size effect in VERTOS II [19] comparing with NEJM trials [4, 5]).

## 3. Are VAPs Cost-Effective? Mortality Reduction

Edidin et al. published a retrospective study on a large Medicare population that showed reduced mortality, a 10% survival benefit in patients undergoing VAPs over NSM, with KP patients having 23% reduced mortality relative risk comparing to VP [47]. Adjusted life expectancy was 85% greater for operated than nonoperated patients, while KP patients had a 34% greater adjusted life expectancy than vertebroplasty patients [3]. Same results were published from another Medicare database; estimated three-year survival rates were 42.3%, 49.7%, and 59.9% for NSM, VP, and KP, respectively. The adjusted risk of death was 20.0% lower for the KP than for the VP group. KP patients had the shortest hospital stay and the highest hospital charges [48]. Operated patients in Taiwan also reported fewer hospital readmissions [49]. A prospective study from UK reports reduced mortality and morbidity one year postoperatively [50]. Another UK study from Svedbom et al., regarding cost-effectiveness analysis, claims that KP is more cost-effective than VP/NSM [51]. Although the hospital/operative room costs are higher with KP [52], the possible mortality reduction from KP may explain those results. However, in a recent study from McCullough et al., the authors state that these striking differences in mortality may be attributed to selection bias; after propensity score matching, VAPs have similar mortality rates with NSM [53], highlighting the need for prospective comparative trials with longer follow-up focusing on mortality as primary end-point.

## 4. VAPs and Kyphotic Reduction/Vertebral Height Restoration

One of the advantages of KP over VP [31–36, 39, 41, 42, 44, 54] or NSM [15–18, 25, 55] as suggested by most authorities is the potential for kyphosis reduction. Only 2 prospective comparative studies claim equivalence between procedures [37, 45], the second one being a nonballoon kyphoplasty. Reduction of kyphosis with KP varies from 3.7° to 8° (mean 4.8°) whereas with VP it ranges from 0.5° to 3° (mean 1.7°) [10]. While some surgeons view that postural reduction is the most critical factor determining kyphotic postprocedural correction [56–58], or claiming equivalent (or superior) results with nonballoon KPs [59, 60], other authors [61] as well as the extensive literature documentation with balloon KPs stresses the fact that balloon inflation also plays a role. Another important issue is whether this degree of kyphotic reduction correlates with clinical improvement. Theoretically, an improvement in spinal alignment and biomechanical behavior of the spine should reduce the flexion moments, relax the paraspinal muscles, and lead to more upright posture, reduced pain, and fewer subsequent fractures [10]. Many authors do not investigate or report this outcome, with others reporting positive [31, 41, 55] or no correlation [18, 32, 35, 62, 63] between restoration of sagittal balance alignment and clinical parameters. Perhaps the strongest indication that reduction does matter is the lately published study from the FREE investigators, on the subset analysis of the radiological surgical parameters; the authors report that patients with higher kyphotic angulation correction had higher QoL improvement [55]. In addition, studies in patients with adult degenerative spinal deformity have shown a strong correlation between sagittal balance correction and surgical outcomes.

## 5. Cement Extravasation

The second advantage of KP over VP is the potential for less cement leaking, as shown by most studies [30, 32, 33, 35, 36, 38, 39, 41, 45, 54], whereas some authors found no difference [29, 37, 40, 42]. Polymethylmethacrylate (PMMA) leakage as shown in meta-analysis ranges from 18.1% in KP to 41.1% in VP [10], with reported rates up to 72% in VP (VERTOS II) [19]. Extravasation rates vary significantly between operators as a result of different reporting techniques, modalities (i.e., Computed Tomography versus X-rays), fracture level, cement volume, or viscosity [10]. Besides from cavity creation in KP (either with balloon or curettes) that allows for low-pressure controlled cement filling, cement viscosity playsa pivotal role [64]; an increasing number of publications report reduced leaking rates with higher viscosity cements and specialized cement delivery equipment or cannulas (i.e., side-opening cannulas that allow for medialized cement injection [65, 66]), even for VPs or nonballoon KPs [59, 60].

## 6. Subsequent Fractures

This is another controversial issue, with some authors proposing that the augmented vertebrae have a different modulus of elasticity/stiffness from the adjacent ones, posing larger forces in the surrounding vertebrae (stress-shielding phenomenon) [67], while others view cement interdigitation as a means of internal fixation and strengthening-restoration of the anterior column that leads to reduced flexion moment to the surrounding vertebral bodies [68]. Our study supports the 2nd notion with NSM leading to a double risk for future fractures (22%) versus VAPs (11%), with no difference between procedures [10]. Most prospective comparative published studies report reduced fractures with VAPs [15–18, 20, 22, 25], same rates [4, 14, 19, 28] or less fractures with NSM [13]. The most well recognized risk factor for adjacent fractures is cement leak into the disk space [69–72]; osteoporosis [25, 69, 72] and kyphotic angle/degree of correction [25, 69] are also being implicated. In our practice, as suggested in the literature, to reduce the possibility of further fractures (especially when bony edema is found on the MRI at adjacent levels), we perform prophylactic augmentation [73, 74].

## 7. Adverse Events

VAPs are generally considered to be safe procedures but can result in rare serious complications [10] especially in the context of published trials from specialized centers [4, 5, 19, 22]. However, with the widespread use of these minimal invasive operations from inexperienced surgeons, catastrophic failures and even deaths are being encountered. Cement has been found literally from top to bottom in the human body, including spinal foramen/canal [13, 32, 35], perivertebral segmental vessels [75], vena cava [76], foot (dorsalis pedis artery), heart [77, 78], and lungs [77, 79, 80]. Penetration of vital structures such as the aorta, pericardium (tamponade) [81], and lungs (pneumothorax) has been also encountered. Cardiovascular events such as desaturation on cement application or hypotension may happen in the operating theater, leading to fatalities in exceptional cases [82, 83]. Pulmonary cement emboli is also frequent and may be encountered in up to one-fourth of patients undergoing VP (VERTOS II) [19], fortunately clinically silent in the vast majority of cases. Infection is a threatening complication, often necessitating corpectomy and cement removal and in a big case series it was calculated to have a 0.5% prevalence with 33% mortality [84]. We believe that with attention to detail and sufficient training, KP and VP are indeed low-risk procedures.

## 8. Cement Types, Characteristics, and Optimal Filling

PMMA has Young's Modulus (measure of stiffness) between 1,8 and 3,1 GPa, is more elastic than human bone (14 GPa for cortical bone), and, because of the stress shielding effect PMMA, might cause osteopenia [85].

In order to address these biomechanical disadvantages, bioactive/bioresorbable cements have been developed which show generally a higher bone affinity-index. Calcium phosphate cements (CPC) are divided into apatite CPCs and the less bioresorbable brushite CPCs and have in general lower mechanical properties than PMMA. Drawbacks of CPC are its lack of macroporosity so that fast bone ingrowth does not take place [85], low viscosity, and injectability of the material [86]. Calcium sulfate cements (CSC) have also been used in kyphoplasty. Perry et al. summarise that CSC are nontoxic, bioabsorbable, osteoconductive, and euthermic but have less stiffness than PMMA [87]. The latter is not necessarily disadvantageous since high stiffness may correlate with increased adjacent level fractures after kyphoplasty.

The volume of cement to be injected in order to achieve optimal results remains a point for debate among experts. Biomechanical studies suggest that small cement volumes (14% vertebrae filling or 3.5 cc in L1) may be adequate to restore stiffness to predamaged levels [88], whereas Belkoff et al. and Molloy et al. report that bigger volumes (16%–30%) are needed to restore vertebral strength and stiffness, respectively [89, 90]. These conflicting results demonstrate that it may be difficult to extrapolate biomechanical data in clinical practice. Smaller amounts of cement (3 cc in thoracic and 6 cc in lumbar) than those needed to restore the height of the vertebra may be enough to achieve resolution of clinical symptoms [91]. However, there is growing evidence that bigger cement volumes correlate with more pain resolution [43] and better restoration of sagittal alignment [57, 92] and in our practice we try to achieve maximum cement filling in a safe manner.

## 9. Timing of Procedure

This is another controversial issue, since early intervention in hyperacute fractures (less than 2-3 weeks) may lead to unnecessary surgery [11], whereas late intervention (after 2-3 months) may compromise results [4, 5, 21, 41] and leave patients in recumbence and agonizing pain. Most authorities advocate a trial of 2–6 weeks trial of conservative treatment before resorting to vertebral augmentation [15, 24, 28, 35, 93]. Our data also indicate that after 7 weeks, clinical outcome is suboptimal and therefore surgery should not be further delayed [94]. Patients that are deemed to have a worse natural course include those suffering from burst or significant collapsed fractures [21, 28, 43], thoracic (or especially thoracolumbar [43]) location [95]. If those risk factors exist, an acute intervention is justified. In summary, in low-risk fractures we would opt for NSM (for 3–6 weeks approximately) and resort to augmentation should pain persists or deformity deteriorates, whereas in higher risk fractures (or intolerable pain) we would operate early [94].

## 10. Unipedicular versus Bipedicular Approach

Pros and cons of the unipedicular approach include less operative time/exposure to radiation and reduced cost versus probable suboptimal kyphotic reduction. However, both biomechanical data [96–98] and clinical series [99–102] suggest that unipedicular procedure is safe and effective. Comparative studies also claim no difference in clinical or radiological parameters [103–106] with the exception of a retrospective study by Chung and coauthors who found same pain reduction but superior kyphosis restoration with bipedicular approach [107]. Only difference may be the smaller cement amount filling in unilateral operations [103, 104], which may be as low as 0.8 cc as seen from our data. Overall, there is no evidence to support superiority of bipedicular VAPs and unipedicular approach should be pursued whenever technically feasible [108]. A bipedicular approach is especially indicated in more severe cases of vertebral body collapse as this typically involves the midportion of the vertebral body, leaving the lateral portions accessible to vertebral augmentation.

## 11. Newer Designs

In balloon KP the inflatable bone tamp is designed to create a cavity where the cement is injected. Various such sets have been developed: Kyphon (Medtronic Inc.), Spasy (Joimax Inc.), AVAmax (Carefusion Inc.), and Ky/Spine (Ackermann Inc.).

In radiofrequency-targeted vertebral augmentation (RV-TVA) the Stabilit system (Dfine Inc.) is utilised via a unipedicular approach and a specialized curved curette. Using radiofrequency activation, an ultrahigh-viscosity cement is prepared and then injected through a cement delivery instrument, in a slow, constant pace, minimizing cement leaking and ensuring uniform cement distribution, in a VP-like manner. Though relatively new, this technique might be superior to balloon kyphoplasty in terms of cement extrusion rates and trabecular bone destruction [59, 60, 109].

The KIVA System (Benvenue Medical Inc.) is a novel kyphoplasty technique where a percutaneously introduced nitinol Osteo Coil guidewire is advanced through a deployment cannula and subsequently a PEEK Implant is implanted incrementally and fully coiled in the vertebral body. The system is subject to the currently running KAST clinical trial. Nonetheless, preliminary results show less amount of cement needed to be injected and lower extravasation rates in comparison to balloon KP [110].

## 12. Future Perspective

Evolution in hardware design and cement properties enables the operator to do the procedure faster and more safely. Unipedicular approach is gaining popularity when feasible, especially with the use of curved curettes; ultrahigh viscous cement, with specialized delivery equipment allow for uniform, controlled, low pressure cement filling. In spite of the early termination of the KAVIAR study due to small patient enrollment, other important RCTs are being anxiously anticipated [CEEP study (KP versus VP), OSTEO-6 (KP versus VP versus NSM), STIC2 (KP versus VP), and VERTOS IV (VP versus SHAM)] that will shed light into the controversial issues highlighted in this review.

## Figures and Tables

**Table 1:** 

Author/year of publication	Baseline characteristics	Pain relief	Disability	QoL	Kyphotic angle/VH	Cement leakage	New fractures	Conclusion
(1) Wardlaw et al. 2009 [22], Boonen et al. 2011 [27] (FREE) RCT	(1) **BKP** (149) versus **NSM** (151) (2) **Acute number** (3) **1 mo, 3 mo, 6 mo, 12 mo**	VAS score **BKP** superior to **NSM**(*P* < .0001)	RM **BKP** superior to **NSM**	**SF-36 ** **BKP** superior to **NSM** **EQ-5D** **BKP** superior to **NSM**	NR	27%, none symptomatic	**BKP**: 33% **NSM**: 25% *P*: NR	**BKP** is superior to **NSM** in acute VCFs in terms of pain and QOL/disability. Differences between groups diminished by 1 year.

(2) Liu et al. 2010 [44] RCT	(1) **BKP** (50) versus **VP** (50) (2) **Acute number** (3) **Postop, 6 mo**	VAS score **BKP** equivalent to **VP**	NR	NR	**BKP** superior to **VP** (*P* < .001)	NR	**BKP**: 2 **VP**: 0	Similar clinical results between **BKP** and **VP**. Better height restoration with **BKP**.

(3) Rousing et al. 2009 [11], 2010 [12] RCT	(1) **VP** (26) versus **NSM** (24) (2) **Acute number** (3) **3 mo, 12 mo**	VAS score **VP** equivalent to **NSM** up to 1 y (*P* = .3)	NR	SF36 **VP** equivalent to **NSM** up to 1 y	NR	NR	**VP**/**NSM**: RR: 1.3	No difference between **VP** and **NSM** in 3 months and 12 months

(4) Voormolen et al. 2007 (VERTOS I) [21] RCT	(1) **VP** (18) versus **NSM** (16) (2) **Acute number** (3) **Postop, 2 w**	VAS score **VP** superior to **NSM** 14/16 pts crossovered from NSM to VP	RM **VP** superior to **NSM**	QUALEFFO Mixed results in the subscores between groups	NR	NR	NR	**VP** is more effective than **NSM** in pain relief and function in the immediate postop period (2 weeks)

(5) Buchbinder et al. 2009 [4] RCT—SHAM group	(1) **VP** (38) versus **SHAM **(40) (2) **Acute number (also subacute)** (3) **1 w, 1 mo, 3 mo, 6 mo**	VAS score **VP** equivalent to **SHAM**	RM **VP** equivalent to **SHAM**	AQoL, QUALEFFO, EQ-5D **VP** equivalent to **SHAM**	NR	37%, none symptomatic	VP: 3 **SHAM**: 4	No difference between **VP** and **SHAM** up to 6 months

(6) Kallmes et al. 2009 (INVEST) [5] RCT—SHAM group	(1) **VP** (68) versus **SHAM **(63) (2) **Subacute number(also Acute and Chronic)** (3) **Postop, 2 w, 1 mo,** 3 mo	VAS score **VP** equivalent to **SHAM** Higher crossover rate in SHAM (*P* < .001)	RM **VP** equivalent to **SHAM** (*P* > .3)	EQ-5D, SF-36 **VP** equivalent to **SHAM**	NR	NR	NR	No difference between **VP** and **SHAM** up to 1 month

(7) Klazen et al. 2010 (VERTOS II) [19] RCT	(1) **VP** (101) versus **NSM** (101) (2) **Acute number** (3) Postop, 1 w, **1 mo**, 3 mo, 6 mo, **1 y**	VAS score **VP** superior to **NSM** (*P* < .0001)	RM **VP** superior to **NSM** (*P* < .0001)	QUALEFFO, EQ-5D **VP** superior to **NSM** (*P* < .0001)	NR	72% had asymptomatic cement leakage	**VP** equivalent to **NSM** (*P* = .44)	**VP** is more effective than **NSM** in acute fractures for at least 1 year.

(8) Endres and Badura 2012 [38]RCT	**BKP** versus UNI **BKP** versus **VP**	All equivalent	All equivalent			**VP** more cement leak		**VP** procedure of choice due to less time and RT (but cement leak)

(9) Vogl et al. 2013 [45] RCT	**KP-CD** (49) versus **VP** (28)				equivalent	**VP** more cement leak	3 in **VP**, 2 in **KP**	**KP-CD** reduces posterior cement leak

**Table 2:** 

Author/year of publication	Baseline characteristics	Pain relief	Disability	QoL	Kyphotic angle/VH	Cement leakage	New fractures	Conclusion
(1) Grafe et al. 2005 [16]/2008 [15]; Kasperk et al. 2005 [18]/2010 [17]	(1) **BKP** (40) versus **NSM** (20) (2) **Chronic number** (3) Postop, 3 mo, 6 mo, 1 y, 3 y	Pain score (0–100) **BKP** superior to **NSM**(*P*: .008 at 1 y, *P*: .02 at 3 y)	EVOS NS difference	NR	**BKP** superior to **NSM** (*P* < .0001)	9%, none symptomatic	**BKP** superior to **NSM** (*P*: .03 at 3 y)	**BKP** reduces pain, new VCFs, and doctor's visits in chronic osteoporotic VCFs for at least 1 year. Both PMMA and CaP equally effective in reducing pain and improving mobility. CaP is better for young pts

(2) Komp et al. 2004 [20]	(1) **BKP** (21) versus **NSM** (19) (2) **Acute number** (3) **6 w, 6 mo**	VAS score **BKP** superior to **NSM** (*P*: NR)	ODI **BKP** superior to **NSM** (*P*: NR)	NR	NR	Poorly reported	**BKP**: 36% **NSM**: 64% *P*: NR	**BKP** is superior to **NSM **

(3) Dong et al. 2009 [31]	(1) **BKP** (20) versus **VP** (18) (2) **NR** (3) **Postop, 3 mo**	VAS score **BKP** equivalent to **VP**	NR BKP improved VC more than VP (*P* < .01)	NR	**BKP** superior to **VP** (*P* < .01)	NR	NR	Both procedures have significant pain relief and improve lung function; **BKP** improves vital capacity more than **VP**

(4) Grohs et al. 2005 [41]	(1) **BKP** (28) versus **VP** (23) (2) **Subacute number** (3) **Postop, 4 mo**, **1 y, 2 y**	VAS score **BKP** superior to **VP** (*P* < .05)	ODI **BKP**: superior to **NSM** (*P* < .05) except from 2 y (NS)	NR	**BKP**: 6% decrease (*P* < .0001) **VP**: No decrease	**BKP**: 22.8%, asymptomatic **VP**: 27.5%	**BKP**: 17% **VP**: 3.5%	In subacute **number BKP** is superior in reducing the kyphotic wedge and pain for 2 y. Disability improvement was superior up to 1 yr

(5) Lovi et al. 2009 [32]	(1) **BKP** (36) versus **VP** (118) (2) **Acute number** (3) **1 mo**, 3 mo, **6 mo, 2 y**	VAS score **BKP** equivalent to **VP**	ODI **BKP** equivalent to **VP**	NR	**BKP** had more restoration than **VP**	**BKP** less extravasation than **VP **(*P* < .05**)**	**BKP**: none **VP**: 3.3%, (*P*: NR)	Similar pain relief and function score **BKP** less cement leakage

(6) De Negri et al. 2007 [30]	(1) **BKP** (15) versus **VP** (18) (2) **Acute and subacute** (3) Postop, 2 days, 1 mo, 3 mo, 6 mo	VAS score **BKP** equivalent to **VP**	ODI **BKP** equivalent to **VP**	NR	NR	**BKP**: NONE **VP**: 33%	NR	There is no difference between two groups in pain relief and disability improvement. Cement leakage occurs in **VP**

(7) Pflugmacher et al. 2005 [34]	(1) **BKP** (22) versus **VP** (20) (2) **Acute number** (3) **Postop**, **3 mo, 6 mo, 1 y**	VAS score **BKP** equivalent to **VP**	ODI **BKP** equivalent to **VP**	NR	**BKP** superior to **VP** (*P* < .05)	NR	NR	**BKP** restore significant body height in acute **number**

(8) Röllinghof et al. 2009 [35]	(1) **BKP** (49) versus **VP** (41) (2) **Acute number** (3) **Preop, postop, 1 y**	VAS score **BKP** equivalent to **VP**	ODI **BKP** equivalent to **VP**	NR	**BKP** equivalent to **VP** in kyphosis reduction **BKP** superior to **VP** in height restoration (*P* < .05)	**BKP**: 22.6 **VP**: 25.5%	**BKP**: 13.2% **VP**: 7.8%	Mean vertebral body height restoration was significantly higher in **BKP.**

(9) Santiago et al. 2010 [37]	(1) **BKP** (22) versus **VP** (20) (2) **Acute number** (3) **1 mo, 6 mo, 1 y**	VAS score **BKP** equivalent to **VP**	ODI **BKP** equivalent to **VP**	NR	**BKP** equivalent to **VP**	**BKP** equivalent to **VP**	**BKP** superior to **VP**(*P*: .01)	No difference between operations

(10) Schofer et al. 2009 [36]	(1) **BKP** (30) versus **VP** (30) (2) **Acute number** (3) **1 d**, last follow-up	VAS score **BKP** equivalent to **VP**	NR	SF36 **BKP** equivalent to **VP**	**BKP** superior to **VP** (*P* < .001).	**BKP** less extravasation than **VP **(*P* < .02**)**	**BKP**: none **VP**: 3.3%	Similar pain relief. and QOL **BKP** less cement leakage and better reduction.

(11) Alvarez et al. 2006 [13]	(1) **VP** (101) versus **NSM** (27) (2) **Fractures >6 w and <1 y** (3) Postop, **3 mo,6 mo,1 y**	VAS score (0–10) **VP** superior to **NSM** up to 6 mo	ODI **VP** superior to **NSM** up to 3 mo **NSM** superior to **VP** after 3 mo	SF36 **VP** superior to **NSM** at 3 mo **VP** equivalent to **NSM** after 3 mo	NR	**VP**: 59.6%	**VP** inferior to **NSM** (*P* < .01)	**VP** is more effective than **NSM** in pain relief and function in the early postop period. No difference was observed after 6 months

(12) Diamond et al. 2006 [14]	(1) **VP** (88) versus **NSM** (38) (2) **Acute number** (3) Postop, **6 w, 6 mo, 1 y, 2 y**	VAS score (0–25) **VP** superior to **NSM** only in 6 w (*P* .004)	Barthel Index **VP** superior to **NSM** only in 6 w (*P* .02)	NR	NR	NR	**VP** equivalent to **NSM**	**VP** is more effective than **NSM** in pain relief and function in the early postop period. No difference was observed after 6 months

(13) Bae et al. 2010 [29]	(1) **BKP** (20) versus **VP** (20) (2) Subacute and chronic **number** (3) 1 mo, 3 mo, 1 y, 2 y	VAS score **BKP** equivalent to **VP** (*P* < .05)	ODI **BKP** equivalent to **VP** (*P* < .05)	SF-12 **BKP** equivalent to **VP** (*P* < .05)	NR	**BKP** equivalent to **VP**	**BKP** equivalent to **VP**	Both **BKP** and **VP** were equally effective in improving pain and disability/QOL

(14) Movrin et al. 2010 [33]	(1) **BKP** (46) versus **VP** (27) (2) Acute and subacute **number** (3) Postop, 1 y	VAS score **BKP** equivalent to **VP**	NR	NR	**BKP** superior to **VP** (*P* < .001).	**BKP** equivalent to **VP**	**BKP** equivalent to **VP**	Both **BKP** and **VP** were equally effective in improving pain and have low incidence of new VCFs. BKP is superior in kyphosis correction

(15) Kumar et al.2010 [42]	(1) **BKP** (24) versus **VP** (28) (2) Subacute **number** (3) 1 w, 3 mo, 10 mo	VAS score **BKP** superior to **VP**	ODI **BKP** superior to **VP** (*P* = .04)	SF-36/EQ-5D **BKP** superior to **VP**	**BKP** superior to **VP**	**BKP** equivalent to **VP**	**BKP** equivalent to **VP**	Both **BKP** and **VP** were equally effective in improving pain, disability, and QOL; BKP yielded superior results maintained until last follow-up

(16) Eidt-Koch and Greiner 2011 [23]	(1) **BKP** versus **NSM** (2) 82 pts		RMD **BKP** superior to **NSM**	EQ-5D **BKP** superior to **NSM**				**BKP** patients improve more than for **NSM** QoL

(17) Li et al. 2011 [39]	(1) **BKP** versus **VP** (96 pts) (2) **Acute number** (3) 12 mo	VAS score **BKP** equivalent to **VP**	**BKP** equivalent to **VP**		**BKP** superior to **VP**	**9% BKP** **35% VP**	3 in **BKP**, 2 in **VP**	similar good outcomes. **BKP** better kyphosis correction and less cement leak

(18) Bornemann et al. 2012 [24]	**NSM** For 6 w and then **BKP** or **NSM**	VAS score **BKP** superior to **NSM**	**BKP** superior to **NSM**					**BKP** was clearly better than **NSM** and should be offered much sooner

(19) Movrin 2012 [25]	(1) **BKP** (46) versus **NSM** (61) (2) **Acute number**	VAS score **BKP** superior to **NSM**			**BKP** superior to **NSM**		**BKP** (6.5%) superior to **NSM (16.4%)**	**BK** superior to **NSM** for pain control kyphosis reduction and adjacent number

(20) Omidi-Kashani et al. 2013 [40]	(1) **BKP** (29) versus **VP** (28) (2) Acute/subacute number	VAS score **BKP** equivalent to **VP**		SF-36 **BKP** equivalent to **VP**		**BKP** equivalent to **VP**	**BKP** equivalent to **VP**	**VP** is preferred due to cost and equivalent results

(21) Lee et al. 2012 [28]	(1) **BKP** (82) versus **NSM** (149) (2) Acute/subacute number. (3) 1 mo, 3 mo, 6 mo, 1 y	VAS score **BKP** superior to **NSM** only in 1st mo and then equivalent	**ODI** **BKP** superior to **NSM** only in 1st mo and then equivalent				8 in **NSM**, 5 in **KP**	Prompt **BKP** should not be indicated if no risk factors exist (age, BMD, BMI, collapse)
